# What is in the black box? The discovery of the sigma factor and the subunit structure of *E. coli* RNA polymerase

**DOI:** 10.1016/j.jbc.2021.101310

**Published:** 2021-10-18

**Authors:** Richard R. Burgess

**Affiliations:** James D. Watson Professor Emeritus of Oncology, McArdle Laboratory for Cancer Research, School of Medicine and Public Health, University of Wisconsin-Madison, Madison, Wisconsin, USA

**Keywords:** *E. coli* RNA polymerase, sigma factor, subunit structure, transcriptional regulation, 2-ME, 2-mercaptoethanol, ATCase, aspartate transcarbamylase, GG, glycerol gradient, PC, phosphocellulose

## Abstract

This Reflections article is focused on the 5 years while I was a graduate student (1964–1969). During this period, I made some of the most significant discoveries of my career. I have written this article primarily for a protein biochemistry audience, my colleagues who shared this exciting time in science, and the many scientists over the last 50 years who have contributed to our knowledge of transcriptional machinery and their regulation. It is also written for today’s graduate students, postdocs, and scientists who may not know much about the discoveries and technical advances that are now taken for granted, to show that even with methods primitive by today’s standards, we were still able to make foundational advances. I also hope to provide a glimpse into how fortunate I was to be a graduate student over 50 years ago in the golden age of molecular biology.

I grew up in Seattle, where my parents allowed me to do chemistry experiments in my bedroom, with only an occasional stink bomb or explosion. I was always curious about how things work, a personal trait I have never outgrown. I attended Caltech as a chemistry major from 1960 to 1964 but found biochemistry more exciting than chemistry. I received a wonderful education with teachers including Linus Pauling, John Roberts, Richard Feynman, Ray Owens, Giuseppi Attardi, Norman Davidson, Bob Edgar, and James Bonner. I even played varsity basketball. I undertook my first research project in my senior year in Robert Sinsheimer’s lab working on the new bacteriophage M13. I loved this experience, and it convinced me to go to graduate school. Having grown up and attended college on the West coast, I decided to head East.

I arrived at Harvard in September 1964, to enter the new graduate program in Biochemistry and Molecular Biology, and began working with James D. Watson, in the Watson-Gilbert research group. Jim had recently been awarded the 1962 Nobel Prize for his discovery in 1953 with Francis Crick of the double-helical structure of DNA. I was delighted to be able to join his lab. Wally Gilbert had been a Physics Professor at Harvard but was attracted to molecular biology, and by 1964, he was fully involved in biological research and had joined Jim Watson to form the Watson–Gilbert research group with shared equipment, research labs, offices, seminars, and the essential daily afternoon tea. By the end of 1965, the graduate students in this group included Ray Gesteland, Peter Moore, Mario Capecchi, Jerry Adams, John Richardson, Dick Roblin, Joan Argetsinger (Steitz), Gary Gussin, Albey Reiner, Jeff Roberts, Jan Pero, Volker Vogt, Roger Hendrix, Bob Kamen, and Nancy Axelrod. I was fortunate to be a part of such an outstanding group of colleagues. The Watson–Gilbert lab was an exciting place to be. I remember vividly that all of us felt we were working in the best molecular biology lab in the world. We all had a large dose of self-confidence (arrogance?) and believed we could find the answer first, no matter who our scientific competitors were.

I started research working in a lab at the opposite end of the third floor of the Harvard Biological Laboratories (the Biolabs) from most of the Watson–Gilbert group. I shared a large room with Matt Meselson’s graduate student Garrett Ihler and three of Guido Guidotti's students, Pierre Henkart, John Pringle, and Steve Rosenberg. This was not a modern lab. In fact, I remember that several of the lab benches were equipped with beautiful old brass hand-cranked centrifuges. In retrospect, these relics of the past seem symbolic of the state of technology in the early 1960’s. The advances in equipment and especially methodology during the next 5 years played a big part in the progress I was to make.

Much of my time those first few months was taken up with the flurry of starting classes, taking cumulative exams and two foreign language exams, learning my way around the Biolabs, playing intramural basketball, attending Boston Symphony Orchestra concerts, and getting to know my fellow graduate student and future wife, Ann Baker.

In early 1965, Jim Watson called me into his office to talk about my research project. He said that John Richardson, who had been working on the *Escherichia coli* DNA-dependent RNA polymerase, had finished his research and was starting to write his thesis. Jim suggested that I determine the subunit structure of the enzyme. He showed me recently published electron micrographs, supposedly of RNA polymerase, that showed an apparent hexagonal shape with a 20 Å hole in the middle (1). He speculated that perhaps the hexagon dissociated into two trimers that reformed the hexagon around a double strand of DNA. “Besides,” he said, “this enzyme plays a central role in transmission of information from DNA to RNA to protein. Anything you find about it is bound to be important.” Jim’s intuition about what was important was legendary. As usual, he was right about its importance and that very significant new knowledge could be obtained by studying this enzyme, but he was wrong about its mode of DNA binding. It later turned out that the photos highlighted an elaborate artifact, caused by a large, contaminating protein later shown to be GroE, which is in fact a heptagon. Nonetheless, his advice set me firmly on the path of studying RNA polymerase and gene regulation that would dominate my future career.

Fortunately for me, the other main labs working on bacterial RNA polymerase (Mike Chamberlin at UC-Berkeley, Wolfram Zillig at the Max Planck Institute for Biochemistry in Munich, Jerry Hurwitz at Albert Einstein College of Medicine in New York, Charles Babinet at the Pasteur Institute in Paris, Audrey Stevens at Oak Ridge National Laboratory) and on transcription (Peter Geiduschek at the University of Chicago/UC-San Diego, Ben Hall at University of Washington in Seattle, Joe Krakow at UC-Berkeley, Andre Sentenac at Saclay in Gif-sur-Yvette, and Robert Khesin in Moscow) all focused on things other than subunit structure at that time, thus creating an opportunity for a beginning graduate student who was slowly developing research skills and maturity to tackle this crucial research problem.

It is important to look briefly at what was known about RNA polymerase and regulation of transcription at this time (early 1965). RNA polymerase activity had been discovered in 1959 by Sam Weiss in the rat liver and soon after by Audrey Stevens in bacteria. Mike Chamberlin, working with Paul Berg at Stanford on *E. coli* RNA polymerase, had published the Chamberlin–Berg purification method in 1962 (2), as well as figuring out the basic reaction conditions for RNA synthesis. A variety of studies quickly established that transcription was specific, made use of a complementary DNA template strand, and proceeded from the 5′ end to the 3′ end of the RNA strand, and initiated with a 5′ triphosphate. It was known that the enzyme itself is large and during transcription forms a large complex with DNA and RNA. Nothing was known about the enzyme’s subunit structure or the components of the transcription machinery or their functions. DNA from the bacteriophage such as T4, T7, ΦX174, and lambda could be used as defined templates, but most often, investigators assayed the enzyme using an inexpensive, commercially available source such as calf thymus DNA. John Richardson had shown by sedimentation studies that the enzyme forms what appears to be a dimer at low salt and dissociates into a monomer at high salt. The size of the protomer, the active form, and the subunit structure were all unknown. It was essentially a large black box.

At that time, the Jacob and Monod model of the lactose operon (the promoter, operator, and repressor), dominated thinking about the molecular biology of transcriptional regulation (3). This elegant model gave a clear idea of how negative regulation could work. A repressor protein would bind to an operator sequence and prevent RNA polymerase from engaging the promotor to initiate RNA synthesis. Ellis Engelsberg’s lab had genetic evidence for a positive regulator of the arabinose operon (4), but no one understood how it worked. The general feeling was that transcription of a gene was “on” unless it was turned “off” by a repressor. A repressed gene could be turned back “on” with a small-molecule inducer that bound to the repressor and prevented its binding to the operator to alleviate transcription repression. Little did I know that my work on the subunit structure of RNA polymerase would lead to a whole new way of thinking about the positive regulation of transcription.

## Purification of RNA polymerase–1965 to 1967

### Early attempts–1965

I set out to purify RNA polymerase from cell lysates. Although *E. coli* RNA polymerase comprises almost 1% of the total cell protein and is quite large and negatively charged, it proved to be a challenge to purify in the 100-mg quantities that I needed for my work. At that time, RNA polymerase had the reputation of being quite unstable, and its purification required working 12 h or more in the cold room. I hated the cold room. The cold rooms in the Harvard Biolabs were small, crowded, and generally filthy. If Wally Gilbert had been working in it, it reeked of cigar smoke. He was the only person I ever saw who could mouth pipette and smoke a cigar at the same time. My first effort to reproduce the Chamberlin–Berg procedure was a disaster, partly due to my inexperience and partly because the batch of protamine sulfate from Eli Lilly that was available to me did not work like the batch used in the Chamberlin and Berg article. I got liters of gelatinous glop that couldn’t be clarified by centrifugation, and I ended up with an enzyme that had a final specific activity lower than that of the crude extract! It was a thoroughly discouraging maiden voyage into the realm of protein purification, and it made it very clear to me that I needed a better purification method. Perhaps this experience is what drove me to continue improving on the purification of this enzyme for the rest of my career, developing at least six successively better methods for purification.

I next tried John Richardson’s method, which involved chromatography on a hydroxyapatite column (5). I had difficulty with my home-made hydroxyapatite, and I still got dismal results. Fortunately, I had many chances to optimize purification protocols because every other week for over a year Wally Gilbert’s technician, Chris Weiss (later Chris Roberts), grew 20 to 30 l of *E. coli* for Wally’s ultimately successful attempts to purify the lactose repressor. Wally took the material that precipitated from the cell extract with 33% saturated ammonium sulfate and left the 33% supernatant for me to use on RNA polymerase preparations. This was a rare luxury that was key to refining my procedures.

In the summer of 1965, I attended the Physiology course at the Marine Biological Laboratory at Woods Hole on Cape Cod for 6 weeks. Jim Watson often sent off early graduate students to summer courses; I suspect to allow him to host visiting scientists to spend time in his lab using the space vacated by the student. While I was gone from Harvard, Lionel and Elizabeth Crawford from Edinburgh came to Jim’s lab to carry out electron microscopy on RNA polymerase and its binding to polyoma virus DNA. On my return, I was shocked to find that they had used up the entire batch of pure enzyme that I had worked months to prepare.

During this time, I was almost the only person in the lab doing protein biochemistry. Most were studying the genetics and molecular biology of viruses and *in vitro* protein synthesis. I was fortunate to have two outstanding mentors in protein biochemistry. The first was Guido Guidotti, a dynamic and irreverent Italian who had been hired at Harvard when John Edsall retired. He was my go-to guy for advice on almost every aspect of protein purification and handling. Later, Klaus Weber arrived as a senior scientist in Watson’s group. Klaus, a friendly and very earnest German, was also extremely experienced in protein chemistry. While I often got very different advice from these mentors, I soon learned that there could be several successful ways to solve a problem. I am greatly indebted to Guido and Klaus for helping me develop my protein biochemistry skills and intuition.

### Enhancing stability of RNA polymerase (and other enzymes)–1965 to 1966

I was always looking for better ways to stabilize RNA polymerase. It did me no good to end a procedure with an enzyme that was impure, inactive, denatured, degraded, or precipitated. At that time, the usual way to keep an enzyme stable was to include a buffer, like Tris, to maintain a pH that is tolerated by the enzyme as well as a chelating agent, such as EDTA, to absorb any contaminating metal ions, such as iron or copper, that could accelerate the oxidation of the protein or inhibit the enzyme. Even so, many enzymes are not very stable in solution, especially RNA polymerase. I found that glycerol and DTT helped tremendously to stabilize it.

I found the stabilizing effect of glycerol pretty much by accident. At that time, I was using 10% to 30% glycerol density gradient centrifugation to fractionate RNA polymerase from smaller proteins. One Friday, I centrifuged three identical tubes, collected fractions that afternoon from two of them, and stored them in the refrigerator. I must have become distracted, because I accidently left one tube out on my bench over the weekend. On Monday, I found the tube, dripped it, assayed it, and found, amazingly, that no activity had been lost. The fact that the enzyme activity had not been lost after sitting at room temperature for 2.5 days suggested that the glycerol was stabilizing the enzyme. From that time on, I used 5% glycerol in all my buffers and included 50% glycerol in my storage buffer, to maintain the stability of the purified material. I had not been aware of earlier work on the protective effect of glycerol (6, 7) because glycerol had not yet been used routinely in protein purification.

About this same time, Mo Cleland at the University of Wisconsin-Madison published an article describing a new reducing agent called DTT, also known as Cleland’s reagent (8). Reducing agents are added to buffers to prevent oxidative damage to proteins. DTT had several advantages over the most commonly used reducing agent, 2-mercaptoethanol (2-ME). It is effective at a lower concentration (0.1 mM compared with 5 mM for 2-ME), is more stable, and, compared with the rotten egg stench of 2-ME, is less volatile so it has almost no odor. DTT became commercially available in 1966, and from then on, I used 5% glycerol and 0.1 mM DTT in all my buffers. This buffer, which I called TGED buffer because of its main ingredients (10 mM Tris, pH 7.9, 5% glycerol, 0.1 mM EDTA, 0.1 mM DTT, 50 mM KCl), made a huge practical impact on my research and in labs across the country. After that, I spent much less time in the cold room.

### Purification using low-salt and then high-salt glycerol gradient centrifugation–1966

**Figure 1:**
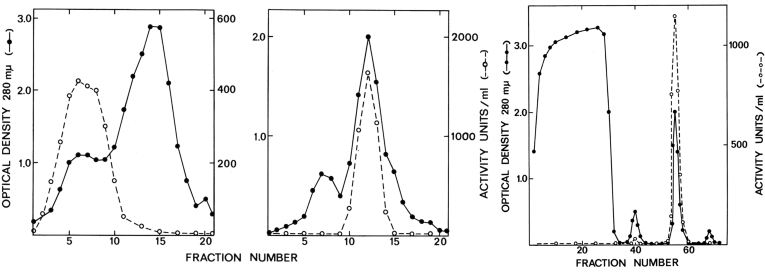
**Two ways to purify RNA polymerase.** Fractionation profiles of low-salt (*left panel*) followed by high-salt (*middle panel*) glycerol gradient centrifugation to produce the GG enzyme, and phosphocellulose column chromatography (*right panel*) to produce the PC enzyme. *Solid lines* are OD_280 nm_, and *dotted lines* indicate the enzyme activity. Reproduced and adapted from Figures 2 and 4 from reference (9) with permission. This research was originally published in the *Journal of Biological Chemistry*. Burgess, R. R. (1969) A new method for the large-scale purification of *Escherichia coli* DNA-dependent RNA polymerase. *J. Biol. Chem.* 244, 6160–6167. GG, glycerol gradient; PC, phosphocellulose.

Normally, one does not do the same fractionation step twice, but in this case, it was very effective. My first new purification procedure took advantage of the property of RNA polymerase to exist as a monomer at high salt concentrations and to form a dimer at low salt concentrations (5). Centrifuging at low salt (about 0.05 M KCl) reveals the dimer at 23S (a measure of its rate of sedimentation that indicated a size of around 900 kDa), which provided an effective means to remove most other proteins that are smaller in size. Fractions containing the polymerase activity were then pooled, the salt increased to 0.3 M KCl, and the protein re-centrifuged in a high-salt glycerol gradient (GG), where it dissociated to a 13S monomer (about 450 kDa) removing protein contaminants that were larger (Fig. 1, left and middle panels) (9). This use of low and high salt sizing steps in a sequential manner gave active, reasonably pure RNA polymerase, referred to as the GG enzyme (later called holoenzyme). However, the GG method was still difficult to scale up to produce the quantity of protein needed at that time for protein chemistry.

### Purification using phosphocellulose column chromatography–1967

In late 1966 and the Spring of 1967, I was fortunate to have Scott Keller, a bright Harvard undergraduate, working with me on some of my experiments. We were trying to develop an even better method to purify large amounts of RNA polymerase. I had been using column chromatography with diethylaminoethyl cellulose, a positively charged column resin that binds negatively charged proteins such as RNA polymerase. However, it also bound other proteins that coeluted with the polymerase, meaning that the purification achieved was not very large. One day in March, I decided to try a column of phosphocellulose (PC), a negatively charged resin containing phosphate groups that binds positively charged proteins. I decided to try my usual pH 7.9 buffer. We prepared a crude extract, carried out a few other steps, and then applied the sample to the column. Most of the protein did not bind and passed directly through the column, the “flowthrough.” We washed the column with a low-salt buffer to remove any remaining unbound material and then eluted with a gradient of increasing KCl concentration and collected fractions. Most of the protein, as measured by UV absorption at 280 nm, was in the flowthrough. One sharp peak eluted rather late in the salt gradient. To our great surprise, this peak had all the RNA polymerase activity. We had removed tremendous amounts of unwanted proteins and obtained an excellent yield of polymerase activity in this one small peak (Fig. 1, right panel) (9). In hindsight, this worked because RNA polymerase bound tightly, presumably because of its positively charged groove where negatively charged DNA would normally bind. PC was mimicking DNA. This turned out to be a very effective purification method that could be scaled up easily. I soon developed this into a useful purification procedure and sent this protocol out to many of my colleagues in the RNA polymerase field. The enzyme purified this way was referred to as the PC enzyme (later called the core enzyme, or as my French-speaking Belgian technician, Anne-Marie Piret, used to say, “Coeur de l’enzyme”).

Little did I know then that the PC method removed a crucial part of RNA polymerase, sigma factor, as I will describe below. I did not realize that the PC enzyme is different from the GG enzyme for two reasons. First, I was assaying my enzyme using commercially available calf thymus DNA as a template, which has nicks and breaks and can be transcribed well by both PC and GG enzymes. Second, I had not started doing sensitive PAGE and staining and therefore had not seen the extra protein band (sigma factor) present in the GG enzyme.

## SDS-PAGE, a huge technical boost to my research

The development of PAGE had a huge effect on protein biochemistry in the mid-1960s. These gels allowed one to easily and quickly assess the purity and also to determine the size of a polypeptide. In 1964, Ornstein and Davis published two highly important articles (10, 11). Until this time, electrophoresis was done in solution, or more commonly, in an anticonvective medium such as a cellulose acetate strip or a gel made of starch (12). Ornstein and Davis showed that one could polymerize acrylamide in a cylindrical glass tube. A sample is loaded on to the top of the gel, and a voltage is applied. After a marker dye nears the bottom of the gel, one removes the gel from the tube and puts it into a stain that diffuses into the gel and binds to the proteins. Soaking the gel in a buffer allows the unbound stain to diffuse out and reveals a series of stained bands. Using buffers during electrophoresis that are compatible with protein structural integrity gives bands representing native, undenatured proteins or protein complexes. This is referred to as a native gel. However, adding a protein-denaturing agent such as 8 M urea to the gel and the loading buffer causes the protein to be denatured and the individual polypeptide chains to separate from each other and migrate independently. This is referred to as a denaturing or urea gel.

In late 1965, I remember Dave Hogness, a well-known biochemist from Stanford, visiting our lab and informally telling us about this new technique. I was eager to try it to see whether RNA polymerase was composed of several different subunits that might be able to be separated by PAGE. I believe I was one of the first people to try PAGE at Harvard. I tried both native and urea gels and found that, yes indeed, I could see several different bands on urea gels. However, there were several problems. First, it was not clear which bands were part of RNA polymerase and which were contaminating proteins. Second, the stain that was commonly used at that time, amido black, was not a very sensitive stain and one needed about 5 to 10 μg of protein in a band to see it. Minor components could not be detected. In late 1966, I read an article by Fazekius de St Groth that reported a new protein stain called Coomassie Brilliant Blue that seemed to be much more sensitive (13). I wrote and asked for some of this stain, and he graciously sent me a sample. I was astounded at the difference! Using this new stain, I could easily see 0.5 μg or less of protein in a band. It was more than ten times more sensitive! All of a sudden, what I thought was relatively pure polymerase did not appear nearly so pure. All my colleagues in the Biolabs who were running gels were eager to use some of my stain solution.

The next year, Shapiro, Vinuela, and Maizel (14) showed that one can use a gel that uses a phosphate buffer containing SDS. SDS is a strong denaturing detergent that binds very tightly to proteins and causes them to unfold and, because of its negatively charged sulfate, become highly negatively charged. In an electric field, these negatively charged, denatured proteins migrate toward the anode, but because they are so highly charged, their mobility through the gel is determined almost entirely by the sizes of the proteins and not by their original charge. This had important consequences. Individual polypeptide chains migrate with a mobility inversely proportional to the logarithm of their molecular weights. This means it is possible to estimate the molecular weight of a polypeptide by comparing its gel mobility to the mobilities of a series of proteins of known molecular weights. I soon ran SDS gels almost exclusively. My enthusiasm for gel electrophoresis led me to create a lengthy “Guide to Gel Electrophoresis” manual that I refined over the next 10 years. Although I never formally published this, I distributed over 2000 copies to colleagues around the world and helped teach many people how to carry out this incredibly powerful technique.

With some effort, I convinced Klaus Weber of the importance of SDS gels in determining the molecular weight of proteins and taught him how to run SDS gels. He was not immediately excited about this method, so I suggested he do an experiment using SDS gels in his graduate biochemistry lab course. He was reluctant until I offered to organize and teach that session. Klaus was at that time sequencing aspartate transcarbamylase (ATCase, an enzyme composed of R and C subunits), and I had the students analyze ATCase along with some molecular weight markers. Results from the gels suggested that the subunits were much smaller than the currently accepted estimates. Klaus said, “See, I told you it didn’t work.” A few months later, he finished the amino acid sequence and discovered that the gels were correct. He became a convert and went on to write with Mary Osborne a very highly cited article (15) about SDS gels and molecular weight determination. He published it in the *Journal of Biological Chemistry* where he thought “protein biochemists would see it.” Keith Dunker and Roland Rueckert at the University of Wisconsin-Madison published a similar study a month later, also in the JBC (16). Both articles confirmed and extended the earlier work by Shapiro, Vinuela, and Maizel and helped establish this technique as a central tool for protein biochemists.

## Subunit structure of RNA polymerase–mid-1967 to early 1968

After I had developed the PC column purification procedure and could prepare large amounts of pure RNA polymerase (PC enzyme), I set out to determine its subunit structure, the original goal of my thesis work. Until that time, most studied proteins that contained more than one subunit had been found to be multimers of identical subunits. A few, such as hemoglobin and ATCase, were known to have two kinds of subunits. I was not aware of any protein complex that had more than two different subunits, although many suspected that the ribosome might contain many different protein subunits. Then, as now, one usually defined a subunit as a polypeptide component of an enzyme or protein complex that copurified with the enzymatic activity through several fractionation steps.

After I saw several bands when analyzing denatured PC enzyme using both urea and SDS gels, I set out to fractionate the polypeptides under denaturing conditions. I succeeded in separating subunits by several chromatography methods. I separated the mixture of what I later called beta prime (β′, 155 kDa) and beta (β, 150 kDa) from alpha (α, 36 kDa) and from omega (ω, 10 kDa) using gel filtration in the presence of SDS. I then separated β′ from β by anion exchange chromatography on diethylaminoethyl cellulose in the presence of 8 M urea (17).

I characterized the separated subunits by amino acid analysis and by cyanogen bromide cleavage patterns. I also characterized the subunits by sedimentation velocity and equilibrium sedimentation under denaturing conditions in the Beckman Model E ultracentrifuge, with the help of another very capable undergraduate, Joanna Hornig. I also determined the sizes of the subunits by SDS gel electrophoresis. I determined the subunit stoichiometry of the PC enzyme by carefully staining and destaining an SDS gel and then quantifying the amount of stain in each band. By dividing the amount of stain in a band by its molecular weight, I determined the relative number of copies of each subunit. All this allowed me to conclude that there are four different subunits in the PC (core) enzyme with the subunit composition of β′βα_2_ω and a molecular weight of ∼390 kDa (17). This result allowed me to proclaim victory (prematurely, it turns out) in my goal of determining the subunit structure and started me thinking about writing my thesis.

Guido Guidotti, a member of my thesis committee, had argued that maybe the subunits I had begun calling α and β'/β were just monomer and tetramer, respectively, of the same subunit. Guido bet me a bottle of fine Italian wine (I recall it was called Lacryma Christi) that they were, but my effort convinced him the subunits were distinct. I collected on the bet, Ann and I enjoyed this delicious wine, and I gained his eventual approval of my thesis.

## Communicating results, setting the stage—January 1968 to August 1968

In the early stages of developing the PC purification, SDS gel results suggested that there were two main subunits, one about 40 kDa and one about 160 kDa. Based on the example of hemoglobin, I named them α and β, respectively. I gave a 10-min talk at the FASEB meeting in Atlantic City in April 1968 where I described the new PC purification method and my evidence for multiple subunits in RNA polymerase. This was my first public talk, and I found it very difficult to cram all I had to say into a mere 10 min. In my abstract, I used the terms α and β but during my talk I reported that recent higher-resolution SDS gels showed that β was really a doublet, which I named β′ and β. Later, I decided that the consistent presence of a 10-kDa band on SDS gels merited a subunit name, and because it was so small, I called it omega (ω).

In June 1968, I attended the Gordon Conference on Nucleic Acids in New Hampton, New Hampshire. I was not scheduled to give a talk, but I told many people about my GG and PC column purification procedures. As was common and collegial in those days, I sent a detailed draft of the PC purification procedure to researchers who requested it, including Ekke Bautz (Rutgers) and Ben Hall (University of Washington). Their labs independently used this method to find sigma.

My research progress was disrupted for 3 weeks in February 1968 by the filming of an ABC TV program called “The Scientist–Race for the Repressor” that took place in the Watson-Gilbert lab. This program focused on the competition between Wally Gilbert and Benno Mueller-Hill on one hand trying to isolate and characterize the lac operon repressor and Mark Ptashne and Nancy Hopkins on the other working to isolate the lambda phage repressor. While this was interesting and often amusing, the omnipresent film crew as well as the huge cables running down the halls and into the cold room to energize Klieg lights were major distractions to research. Once, while a bacterial air shaker filled with flasks of swirling, colored liquids was being filmed, my wife Ann told the film crew that this was unrealistic and that scientists would know it was staged. They replied, “Scientists do not have color TVs.” I had the honor of being the “typical graduate student” and responding to Wally’s questions about my SDS gels of RNA polymerase displayed on a light box. The best part was the huge lab party that Jim held to celebrate the airing of the program and our film debuts.

In January 1968, I had received a Helen Hay Whitney Fellowship for my upcoming postdoctoral training with Alfred Tissieres at the Institute of Molecular Biology in Geneva, Switzerland. I was eager to shift from a $2400/year graduate stipend to a $7000/year postdoc stipend; however, I could not start until after defending my thesis. As a result, I was trying hard to focus on writing. A rapid series of events conspired to delay my thesis by about 5 months but resulted in the most exciting and productive time in my scientific career.

## Discovery of the sigma factor–September 1968 to January 1969

In September 1968, Andrew Travers arrived from Cambridge, England, as a new postdoc in the Watson Lab. He was a soft-spoken, creative Englishman looking for a project. Because it was a large lab, I did not pay much attention to him at first, not knowing that he would play a very important role in the coming months. My fellow lab mate in room 388, Jeff Roberts, was studying transcription of lambda phage DNA *in vitro*. For at least a year, he had been using my GG enzyme for his transcription studies, and it had worked very well. During September, he ran out of the enzyme and asked me for some more. I was very proud of my pure and well-characterized PC enzyme and gave him a big portion. This PC enzyme was very active on calf thymus DNA, but Jeff shocked me by announcing that it was not active in transcribing high-quality, intact phage lambda DNA. This was unexpected because I considered it to be the best enzyme I ever made.

In early October, based on Jeff’s concerns and some preliminary *in vitro* transcription experiments by Andrew Travers showing that phage T4 DNA also was not transcribed by PC enzyme, I decided to do one last RNA polymerase preparation before writing my thesis. I needed to understand the difference between the GG and PC enzyme. I planned to prepare a fresh batch of the GG enzyme and pass it over a PC column to see whether something that is required for activity on intact phage DNA is lost during this step. I would do all this very carefully and monitor each step and fraction with enzyme assays and SDS gels. Anne-Marie Piret, my technician, had been working full time with me since the previous November, and she and I were at the top of our games. I had spent almost 4 years making mistakes and learning lessons the hard way and was now well prepared to design, execute, and analyze several major experiments a day.

**Figure 2:**
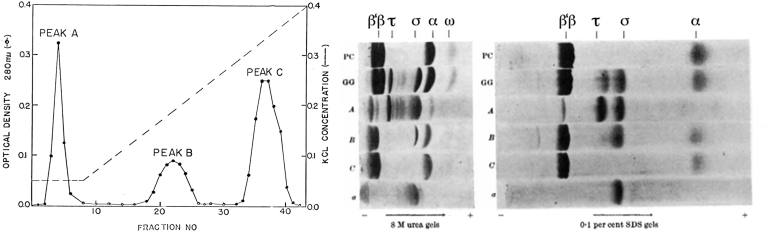
**Phosphocellulose chromatography of the GG enzyme (*left panel*) and PAGE of phosphocellulose samples on 8 M urea gels (*middle panel*) and 0.1% SDS gels (*right panel*).** Reproduced and adapted from Figures 2 and 3 from reference (18) with permission. This research was originally published in *Nature*. Burgess, R. R., Travers, A. A., Dunn, J. J., and Bautz, E. K. F. (1969) Factor stimulating transcription by RNA polymerase. *Nature* 221, 43–46. GG, glycerol gradient.

**Figure 3:**
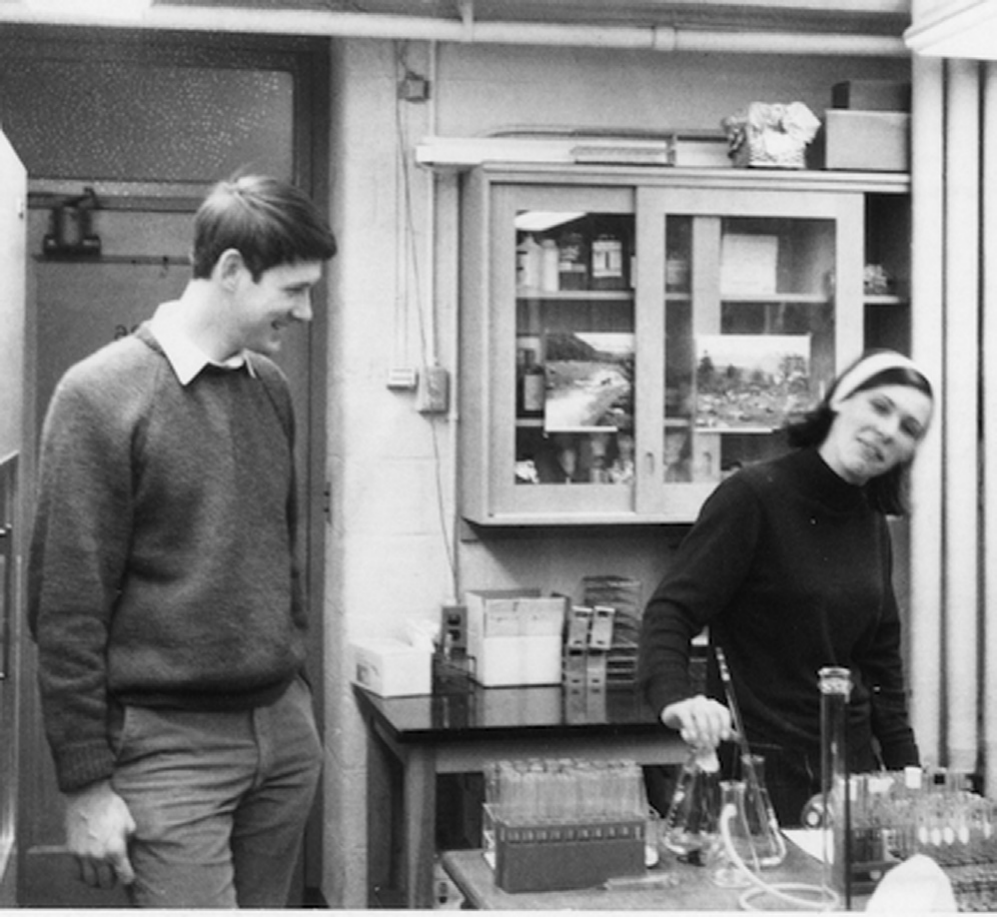
**Dick Burgess with Anne-Marie Piret, Harvard, 1968**.

On October 24, we ran a PC column on the freshly made GG enzyme (Fig. 2) (18). On October 25, the assay results showed that enzyme activity eluted in its usual peak was active on calf thymus DNA but not on intact T4 phage DNA. Most importantly, the activity on T4 DNA was restored when the flowthrough from the PC column was added back to material from the PC enzyme peak. The flowthrough fraction contained a novel stimulatory factor! Now, we knew the reason for the inactivity of PC enzyme on T4 and lambda DNA. Apparently, when the GG enzyme bound to the PC column, this stimulatory factor was released. The flowthrough protein was not active on either template by itself. Andrew and I began working together full time on characterizing the system. Anne-Marie worked very hard running numerous columns, gels, and assays (Fig. 3). Fractions from the PC enzyme peak showed the normal RNA polymerase subunits on SDS gels, β′, β, α, and ω (Fig. 2, right panel). However, the flowthrough material contained two additional bands, one about 80 kDa and another one about 110 kDa. We quickly showed by GG centrifugation that the 80-kDa protein was the stimulatory factor. We called this the “sigma factor” for “stimulate” or “start” because we suspected it was an initiation factor. For a short time, we called the 110-kDa band “tau” because we thought it might be a termination factor. That turned out not to be true. Jeff Roberts, barely 25 feet away across the room, was just in the process of discovering the real termination factor, rho (19). We also showed, using native gels, that the sigma factor could bind to the PC enzyme and reconstitute the GG enzyme. In about a week, we had made huge strides in defining and characterizing the system. These were very heady times.

I had trouble sleeping those first few nights. I was very excited over the realization that we had made a major breakthrough in understanding RNA polymerase function and specificity. It now seemed obvious that RNA polymerase core could not bind and correctly and efficiently initiate transcription on an intact DNA template without a positive factor to direct it to a promoter. Sigma seemed to be providing the specificity for promotor binding. It seemed clear that at least one positive transcription factor, sigma, existed, and we knew how it worked. Perhaps, there would be multiple sigma factors, each with a different promoter specificity? Perhaps, this was a mechanism for the general regulation of classes of genes? We had much exciting work to do.

On October 30, we heard from Ray Gesteland, who had just stopped by after giving a seminar at Rutgers, that John Dunn, a graduate student with Ekke Bautz at Rutgers, had very similar results. John was studying T4 phage transcription *in vitro* and was using my method for making the PC enzyme. He had also discovered that the PC enzyme did not work well on an intact template.

At Jim Watson's suggestion, we called Ekke and agreed that our two labs would write up our work as one article and submit it to Nature. On Tuesday, November 5 (I remember it was the election day when Nixon beat Humphrey), Ekke and John came to Harvard and we planned the article. It ended up containing mostly my and Andrew's data because they were clearer and more complete, but it was definitely a joint effort. Andrew and I continued to actively pursue our investigation of sigma and its role in transcription initiation. The manuscript was received at Nature on December 2, a mere 5 weeks after our initial discovery. On January 4, 1969, the article, “Factor Stimulating Transcription by RNA Polymerase” appeared in Nature (18).

I was amazed at the publicity our publication generated. There were several long “News and Views” columns published in Nature commenting on the discovery and even a feature article in the Boston Globe.

In retrospect, the excitement created by sigma overshadowed my subunit work, which had actually resulted in the discovery of one of the first protein complexes containing three or more different subunits. RNA polymerase was one of the first characterized molecular machines that now are known to be ubiquitous, central players in transcription, replication, translation, signal transduction, protein degradation, mRNA splicing, apoptosis, and much more.

Many people have asked me why Jim Watson’s name was not on the article. Jim never put his name on articles from his lab unless he did some of the work himself. As a result, I got much more notoriety as the first author than I normally would have. Jim was very pleased by our results and describes in his 2007 book “Avoid Boring People” (20) how much satisfaction he got giving a talk in late 1968 about the discovery of sigma and the subunits of RNA polymerase to the Biochemistry Department at Stanford. I vaguely remember him asking me for copies of my slides, but I had no idea he had even given this talk until 38 years later when he asked me to read a draft of his book. I am glad he was able to take pride in our work. Without his vision, encouragement, and creation of an outstanding collaborative environment, this work would not have been possible.

In November 1968, I talked to Rich Losick, a new Harvard Junior Fellow in the Biolabs, during lunch one day. We were both members of the Senior Common Room at Leverett House, an undergraduate residence hall. When I told him what I had found, he got very excited about the possibility of sigmas affecting transcription of *Bacillus subtilis* phage in sporulating in *B. subtilis* based on research by his friend Linc Sonenshein at Massachusetts Institute of Technology. Rich decided to focus his research on this, and he became a pioneer in understanding the changes in gene regulation that occur during *B. subtilis* growth and sporulation, especially changes due to appearance and disappearance of multiple sigma factors. Much of Rich’s story of this time is found in his 2015 JBC Reflections article (21).

To my knowledge, there was no working model about gene regulation in eukaryotes at that time. I do not recall thinking much about eukaryotic transcription, except to assume that the process was probably quite similar to that in bacteria. In December 1968, just after our sigma discovery, I went home to Seattle for Christmas holidays and visited the Genetics and Biochemistry Departments at the University of Washington to talk to Ben Hall and others as I was being considered for a faculty position. While there, I spent an hour talking to one of Bill Rutter’s graduate students, Bob Roeder, about his recent work on eukaryotic RNA polymerase. He had just separated the enzyme activity into three peaks by ion exchange column chromatography (22, 23). It later turned out that these three peaks were RNA polymerases I, II, and III, responsible for synthesis of ribosomal RNA, mRNA, and tRNA, respectively. The year 1968 was very good for RNA polymerase research.

## Impact of sigma discovery and aftermath

In February 1969, I defended my PhD. thesis, “Subunit Structure of *E. coli* RNA Polymerase” before a committee consisting of Jim Watson, Wally Gilbert, Guido Guidotti, Charlie Thomas, and Howard Berg. I remember getting a nice note from David Baltimore, then at MIT, in which he congratulated me on the discovery of sigma and included a small article packet of white granular powder labeled PC (which actually stood for pure cane sugar). In March, I traveled to Europe to give talks about my recent work for Benno Mueller-Hill's Phage Course in Cologne, Wolfram Zillig in Munich, Alfred Tissieres in Geneva, Francois Gros in Paris, Heinz Schaller in Tubingen, and Guido Hartmann in Wurzburg. It was thrilling to be greeted as a visiting dignitary.

When I returned to the Biolabs from this trip, Jim asked me if I had told everyone about Jeff’s discovery of the termination factor rho. I said, “No, it’s his work and his story to tell, not mine.” Jim was mad at me (the only time I remember this happening) for my failure to communicate this new information. While I felt comfortable with my actions, it was clear that Jim felt very strongly that exciting science should be shared with others openly and immediately.

During late 1968 and early 1969, Andrew and I continued to study the details of sigma action. It was very clear that the enzyme lacking sigma (PC enzyme, or as we were beginning to call it, “core” polymerase) was capable of RNA synthesis but requires sigma to form the holoenzyme that binds to specific DNA sites, the promoters of Jacob and Monod. It had not escaped our attention that it was very plausible that there could be several sigmas that recognized different types of promoters. We hinted at this multiple sigma hypothesis in our first article (18):

*“If sigma itself determines the specificity of initiation, the interesting possibility arises that several similar factors could exist, each with a specificity for a different type of initiation site.”*

This hypothesis turned out to be true.

In May, Andrew and I published a second article in Nature, “Cyclic Re-use of the RNA Polymerase Sigma Factor” (24). This established a clear mechanism for the involvement of sigma in selective binding to promoters and efficient initiation of transcription, followed by the subsequent release of sigma from the transcription complex soon after initiation of the RNA chain. This “sigma cycle” explained how sigma could be reused and was therefore catalytic in its function. It also provided a mechanism by which a new type of sigma, if it existed, could bind to core polymerase and direct the polymerase to a new class of promoters. This strengthened the multiple sigma hypothesis.

I was invited to present a talk in April at the Federation meetings and asked to write a review article with Andrew. This article, “*E. coli* RNA Polymerase: Purification, Subunit Structure, and Factor Requirements” (25) concludes with the following paragraph:

*“A mechanism for general positive control. Since sigma is able to stimulate catalytically the initiation of RNA from specific genes, . . . it appears that each factor enables the core polymerase to recognize a different class of promoter sites on DNA. This amounts to a system of positive control that may be a fundamental mechanism of regulating RNA synthesis in bacteria. These positive control elements, acting at the level of initiation, would turn on whole classes of RNA simultaneously and would thus act as a coarse control. For a given sigma factor, the level of transcription of genes under its control could be regulated by its relative abundance, stability, and binding affinity for core polymerase, all of which variables could affect the ability of the factor to compete for available core polymerase.”*

**Figure 4:**
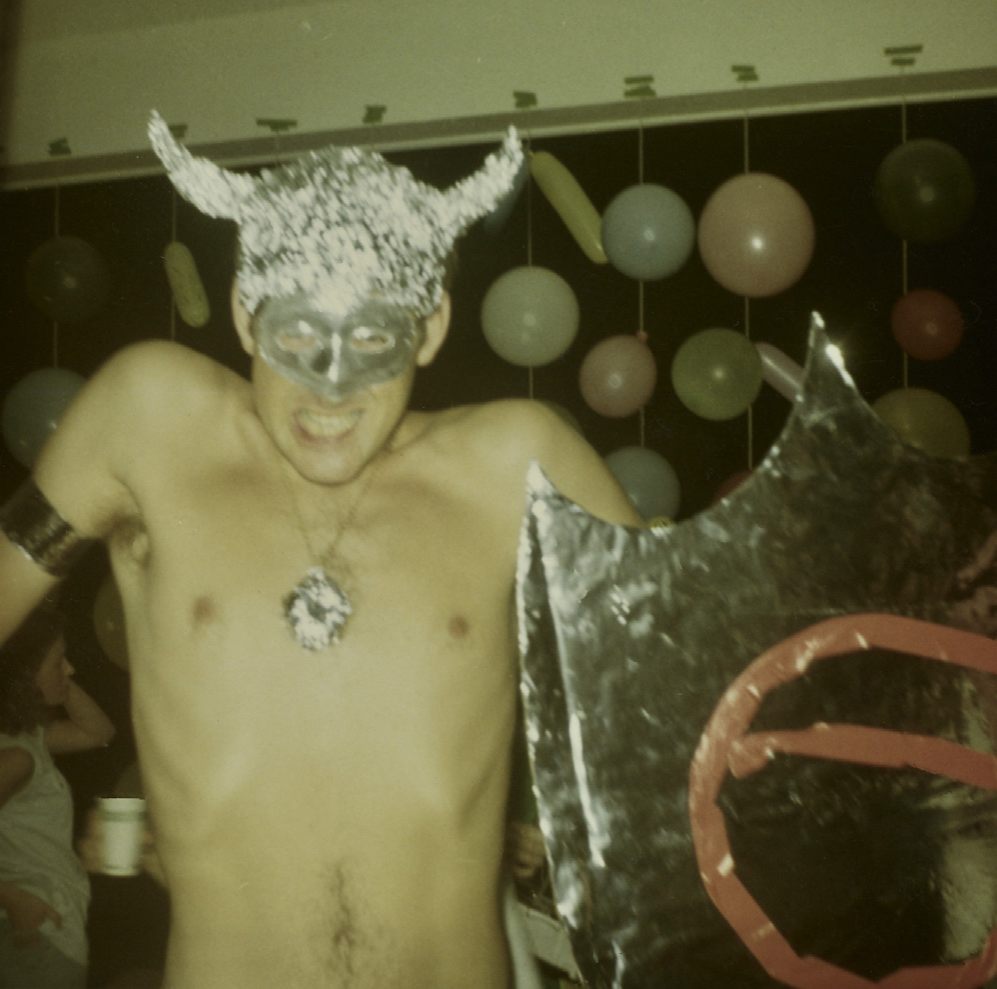
**Dick Burgess, dressed as Sigmund from a Wagnerian opera, at the Animal Virus Course costume party, Cold Spring Harbor Laboratory, July 1969**.

That summer, I accompanied my wife Ann when she attended the Animal Virus Course at Cold Spring Harbor Laboratory on Long Island. In addition to driving to the nearby town of Huntington to buy donuts for the course each morning, I worked on converting two of my thesis chapters, one focusing on the details of my *E. coli* RNA polymerase purifications (9) and the other on the separation and characterization of its subunits (17), into two articles that were published in the JBC in November 1969. I also started working on a review for Annual Reviews of Biochemistry on RNA Polymerase that I finished during my postdoctoral research in Geneva, Switzerland, in Alfred Tissieres’ lab (26). One of the highlights of that summer at Cold Spring Harbor was watching the Apollo 11 moon landing on TV. There was also a wild costume party associated with Ann’s course in which I dressed up as the Wagnerian figure, Sigmund, complete with aluminum foil Teutonic horned helmet and a shield emblazoned with a huge Greek letter sigma (Fig. 4). Later that summer, we returned to Cambridge, and Ann defended her thesis with David Denhardt on the proteins of the bacteriophage ΦX174 (27). We left Harvard in August and, after a trip around the country to visit relatives and do a little job hunting, began our postdoctoral fellowships in Geneva.

**Figure 5:**
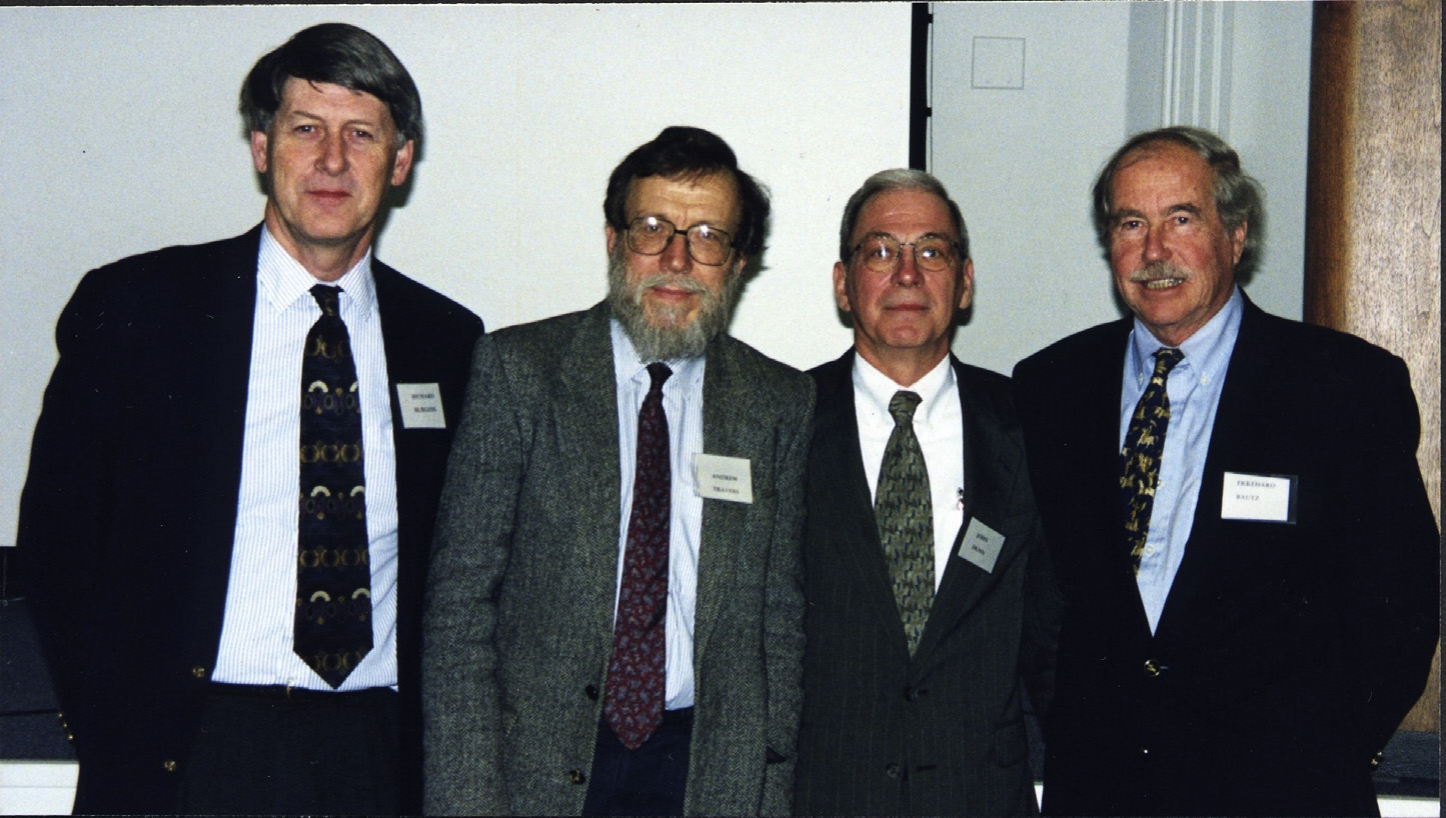
**Dick Burgess, Andrew Travers, John Dunn, and Ekke Bautz at the 30th Anniversary celebration of the discovery of sigma at the Waksman Institute, Rutgers University, December 1999**.

**Figure 6:**
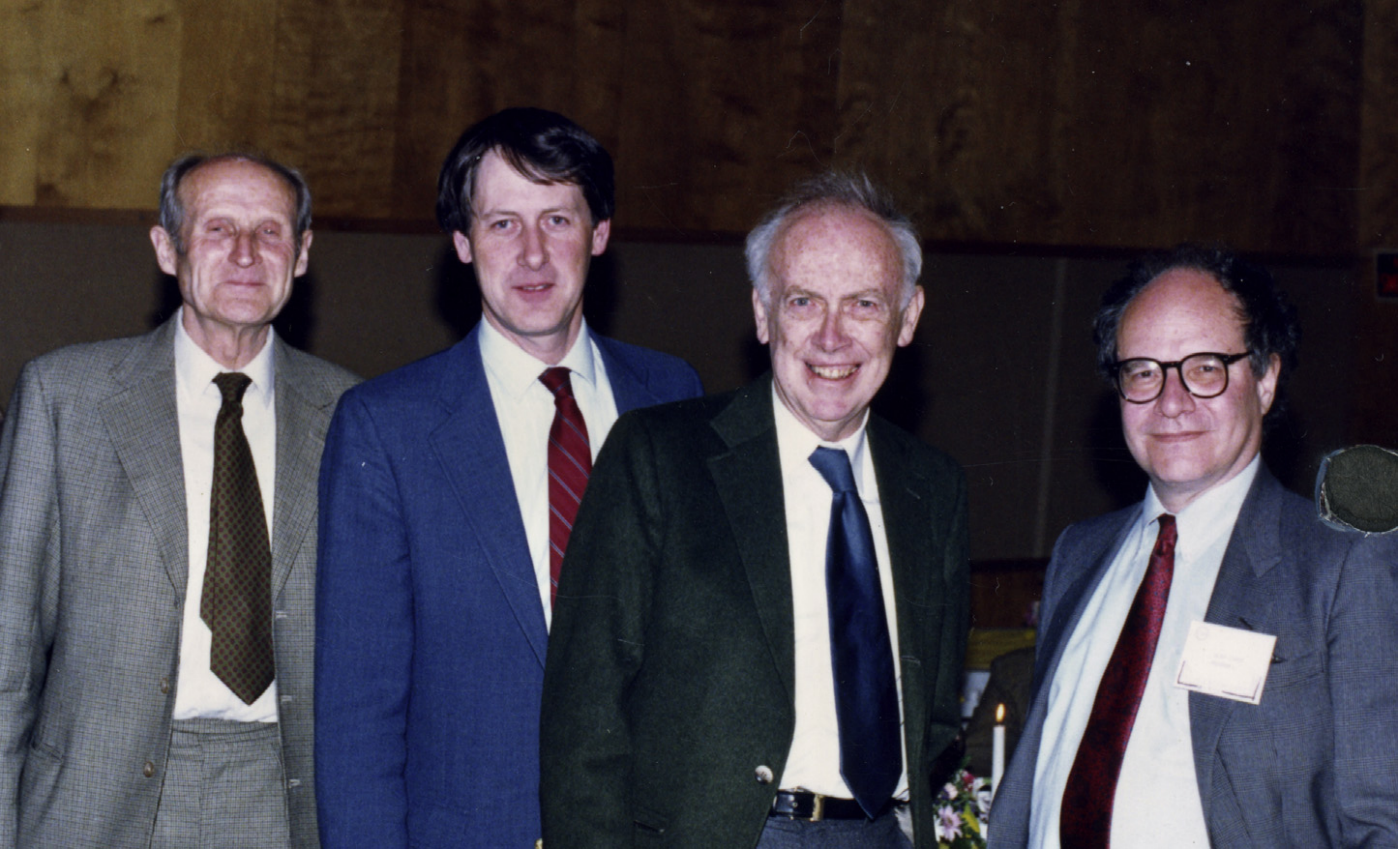
**Alfred Tissieres, Dick Burgess, Jim Watson, and Wally Gilbert, Cold Spring Harbor, 1988**.

**Figure 7:**
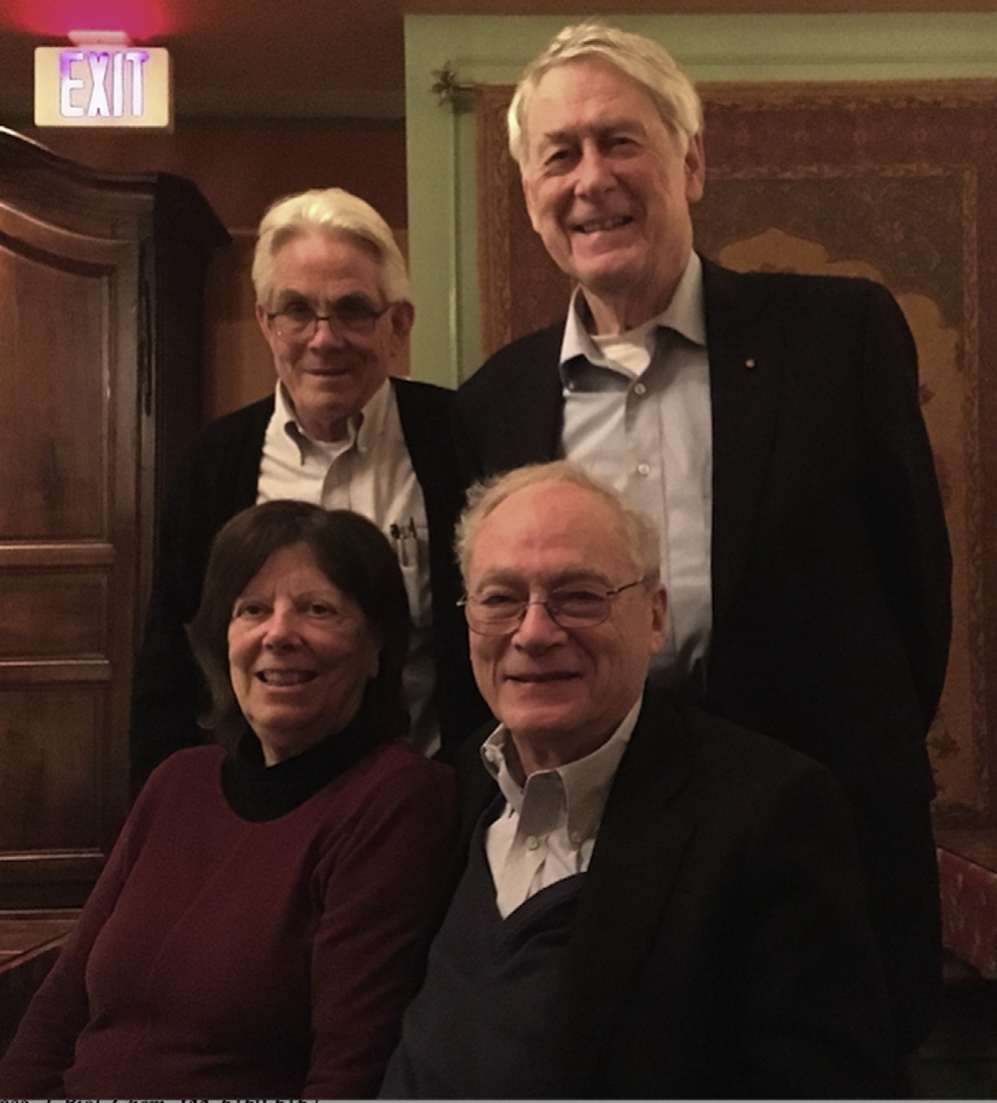
**Jeff Roberts, Dick Burgess.** Jan Pero, and Rich Losick at the 50th Anniversary celebration of the discovery of sigma at the Waksman Institute, Rutgers University, December 2019.

Photographs in Figure 5, Figure 6, Figure 7 show some of the major actors in the story I have just told.

### Other contemporaneous results–I was lucky

While I have focused on what was happening at Harvard and Rutgers, the RNA polymerase world had not stood still. Several other labs had made major progress. In particular, Ekke Fuchs and Peter Palm in Wolfram Zillig’s lab had also discovered the subunit structure of RNA polymerase. However, their article was rejected because the reviewer could not believe that protein subunits could be as large as 150 kDa! In early 1969, I received a courtesy copy of this unfairly rejected manuscript and realized how close I had come to being scooped.

Joe Krakow at Hunter College also independently discovered sigma. He was studying RNA polymerase from the bacterium *Azotobacter vinelandii*. He found that mixing the enzyme with RNA and then electrophoresing it on a nondenaturing polyacrylamide gel released a protein that migrated faster than the rest of the enzyme (28). He originally named this protein gamma, but after our Nature article came out, he graciously renamed it sigma. I suspect that binding to RNA may be similar to binding to PC in releasing sigma from the core.

Gordon Hager, a graduate student with Ben Hall in Seattle, was also studying *in vitro* transcription of phage T4 and had started using the PC enzyme. He was well on his way to independently discovering sigma when our first Nature article appeared. A number of articles were published later in 1969 and 1970 from other labs (including those of Ekke Bautz, Joe Krakow, Mike Chamberlin, Ben Hall, Wolfram Zillig, Andre Sentenac, and Robert Khesin) that added considerably to our growing knowledge of bacterial RNA polymerase and transcription factor sigma.

The excitement and press coverage of sigma created sort of a “Sigmania” where several research groups incorrectly interpreted their results as new sigma-type factors. However, additional legitimate sigma factors were eventually discovered. In particular, Rich Losick and Jan Pero at Harvard were studying the role of sigmas in phage infection and sporulation in *B. subtilis*. Jan and her student Tom Fox found the first alternative sigmas, those involved in phage SP01 late transcription, in 1974 (29).

I want to be explicit that work on sigmas is only part of the transcriptional activator story. Jack Greenblatt and Bob Schleif were able to demonstrate AraC function *in vitro* (30), and Bob went on to show that the AraC protein of Engelsberg was indeed a positive transcription factor that increases the binding of RNA polymerase to the arabinose operon promoter (31, 32). In 1970, soon after our sigma work, Geoffrey Zubay discovered a new protein, cyclic AMP receptor protein or catabolite gene activator protein, that mediates the classical glucose effect and is needed for high-level expression of the lac operon and many other genes (33). Since then, investigators have identified hundreds of nonsigma transcription factors in *E. coli* alone that affect transcription of specific operons. Most of these factors bind to specific sites on DNA and interact with RNA polymerase holoenzyme to allow the latter to engage specific promotors and transition to an open promoter complex in a way that is often modulated by a small-molecule effector.

## Epilogue

Ultimately, seven different sigmas were discovered in *E. coli*. The sigma factor we discovered is now called sigma70 or sigmaD and is considered to be the housekeeping sigma that is needed for transcription of the majority of *E. coli* genes. Alternative sigmas allow coordinate transcriptional regulation of specialized groups of genes involved in the heat shock response (sigma32), flagellar synthesis (sigmaF), adaptation to starvation and stationary phase (sigmaS), nitrogen (sigmaN/sigma54) and iron (sigmaFecI) metabolism, and response to extreme temperatures (sigmaE) and extracytoplasmic events (sigmaE and sigmaFecI).

It took another 50 years to fill out our understanding of the structure and function of the various parts of the transcription machinery. Major progress has been made in my lab in McArdle Laboratory for Cancer Research at the University of Wisconsin-Madison and in many labs around the world. Below are some of these areas of progress, illustrated in many cases by representative work from my lab, including some of the method developments that made this research possible.

We continued to find new ways to purify RNA polymerase and sigma. Postdoc Jerry Jendrisak and I popularized the use of PEI precipitation in RNA polymerase purification (34, 35, 36, 37), and postdoc Peter Lowe optimized sigma purification and more thoroughly characterized sigma physical properties (38). Specialist Dayle Hager and I developed a purification of microgram amounts of sigma70 and other proteins by elution from SDS gel bands and renaturation (39, 40). My first two graduate students, Jackie Miller and Edmundo Calva, worked to determine how one could better characterize initiation and termination specificity during *in vitro* transcription of phage DNA (41, 42). In an extensive collaboration with UW-Madison biochemist Tom Record, we studied the biophysical chemistry of core and holo RNA polymerase binding to DNA and the aggregation of core and holo as a function of ionic conditions (43, 44). Harlee Strauss from Tom’s lab and my graduate student Becky Boston developed a beautiful nitrocellulose filter-binding method to detect selective holoenzyme binding to restriction fragments containing promoters (45).

Carol Gross joined my lab as a postdoc in 1973 and later became a senior scientist before she became a faculty member in the UW-Madison Department of Bacteriology in 1981. My lab’s productivity during the mid-70s to early 80s was largely due to her creativity, vision, and technical and interpersonal skills. She was instrumental in using temperature-sensitive mutations in sigma70 to map its gene, rpoD (46). This led to the cloning onto a transducing phage and sequencing of the rpoD gene in collaboration with UW-Madison geneticist Fred Blattner and subsequently to the sequencing by my postdoc Zach Burton and student Bruce Erickson of the complex operon that includes genes for the ribosomal protein S21, sigma70, and DNA primase (47, 48). Mike Gribskov, a graduate student in the lab with excellent laboratory skills as well as expertise in the newly emerging area of computer-based sequence analysis, constructed a recombinant strain of *E. coli* that overproduced sigma70 and used it to purify sigma70 in amounts that had not previously been possible (49). Furthermore, he used amino acid sequence alignment of the emerging diverse collection of bacterial and phage sigma factors to recognize clear sequence homology among them and to identify regions of high conservation that helped define the key functional regions of sigmas (50). Graduate student Dan Gentry cloned, sequenced, overproduced, purified, and studied the omega subunit (51, 52, 53). Graduate students Lam Nguyen, Debbie Jensen, and Larry Anthony went on to overproduce, purify, and study other *E. coli* sigmas (54, 55, 56, 57, 58).

Senior scientist Nancy Thompson, who has played a major role in ensuring the smooth functioning of my lab since she joined it in 1985, identified special “polyol-responsive” monoclonal antibodies that could be used to gently immunoaffinity purify RNA polymerases and transcription factors (59, 60, 61, 62, 63, 64, 65). Graduate students Lin Rao, Scott Lesley, Terry Arthur, Brad Pietz, and Larry Anthony used a variety of techniques, including “ordered fragment ladder far westerns,” to identify and study the regions where the core and sigma interact (66, 67, 68, 69, 70, 71). Graduate student Kai Zhao determined the regulons (genes whose promotors are recognized by and coregulated by a given sigma) for *E. coli* sigma32, sigmaF, and sigmaN (72, 73, 74). Postdoc Veit Bergendahl and graduate student Bryan Glaser used luminescence resonance energy transfer assays to determine the binding affinity of several sigmas for core as a function of solution conditions (75, 76, 77). Many other research groups contributed to our understanding of sigmas from *E. coli* and other bacteria and bacteriophage and how the level of each of the active sigmas is regulated in response to environmental stresses, during development, and during viral infection. Finally, work in many laboratories on the three-dimensional structures of core RNA polymerase, holoenzyme, and holoenzyme bound to a promotor and during initiation has revolutionized our understanding of the structure and mechanism (78). Overviews of this research on sigmas and RNA polymerase can be found in several excellent recent reviews (78, 79, 80, 81, 82, 83). Jeff Roberts has recently published his related historical perspective (84).

Technical advances and early adoption such as improved protein stabilizers, unconventional use of PC chromatography, and PAGE, especially SDS gels, made a huge impact on my progress during my graduate research career. As I established my research program at UW-Madison in the Fall of 1971, I continued to focus on better understanding the transcription machinery, RNA polymerases, and regulatory factors. But I also was keenly aware that developing a new technique or significantly improving an existing technique for use in our polymerase research could have a broad impact in many other fields that use protein biochemistry. As a result, I spent significant time, resources, and creativity on method development in the area of protein purification and characterization. An enjoyable and rewarding phase of my career has been in disseminating and teaching these methods to new generations of protein biochemists through organizing meetings, editing books (36, 85, 86), mentoring students, formal course teaching (23 years of teaching the Cold Spring Harbor Protein Course), writing reviews (87, 88), and reviewing manuscripts (as Editor-in-Chief of the journal *Protein Expression and Purification*).

The last 50 years have witnessed an explosion of research activity around the world on the machinery of transcription and transcriptional regulation in bacteria, archaea, and eukaryotes. The incredible complexity that has been revealed is astounding. I was fortunate to have entered the field in simpler times when we merely wanted to know what was in the black box called RNA polymerase. I was also fortunate to have been both a player and a spectator in that exciting time when molecular biology, biochemistry, and genetics combined with new technical capabilities to produce a truly wonderful and illuminating time in biological science research.

## Conflict of interest

The author declares that he has no conflicts of interest with the contents of this article.
